# Dihydromyricetin Protects Against Gentamicin-Induced Ototoxicity via PGC-1α/SIRT3 Signaling *in vitro*

**DOI:** 10.3389/fcell.2020.00702

**Published:** 2020-07-28

**Authors:** Hezhou Han, Yaodong Dong, Xiulan Ma

**Affiliations:** Department of Otolaryngology Head and Neck Surgery, Shengjing Hospital of China Medical University, Shenyang, China

**Keywords:** dihydromyricetin, aminoglycosides, ototoxicity, PGC-1α, sirtuin 3, reactive oxygen species

## Abstract

Aminoglycoside-induced ototoxicity can have a major impact on patients’ quality of life and social development problems. Oxidative stress affects normal physiologic functions and has been implicated in aminoglycoside-induced inner ear injury. Excessive accumulation of reactive oxygen species (ROS) damages DNA, lipids, and proteins in cells and induces their apoptosis. Dihydromyricetin (DHM) is a natural flavonol with a wide range of health benefits including anti-inflammatory, antitumor, and antioxidant effects; however, its effects and mechanism of action in auditory hair cells are not well understood. The present study investigated the antioxidant mechanism and anti-ototoxic potential of DHM using House Ear Institute-Organ of Corti (HEI-OC)1 auditory cells and cochlear explant cultures prepared from Kunming mice. We used gentamicin to establish aminoglycoside-induced ototoxicity models. Histological and physiological analyses were carried out to determine DHM’s pharmacological effects on gentamicin-induced ototoxicity. Results showed DHM contributes to protecting cells from apoptotic cell death by inhibiting ROS accumulation. Western blotting and quantitative RT-PCR analyses revealed that DHM exerted its otoprotective effects by up-regulating levels of peroxisome proliferator activated receptor γ-coactivator (PGC)-1α and Sirtuin (SIRT)3. And the role of PGC-1α and SIRT3 in the protective effects of DHM was evaluated by pharmacologic inhibition of these factors using SR-18292 and 3-(1*H*-1,2,3-triazol-4-yl) pyridine, respectively, which indicated DHM’s protective effect was dependent on activation of the PGC-1α/SIRT3 signaling. Our study is the first report to identify DHM as a potential otoprotective drug and provides a basis for the prevention and treatment of hearing loss caused by aminoglycoside antibiotic-induced oxidative damage to auditory hair cells.

## Introduction

Oxidative stress results from the perturbation of cellular redox balance (Kandola et al., 2015) caused by excessive levels of reactive oxygen species (ROS) that exceed antioxidant defense mechanisms, leading to the destruction of cellular structures and cell death (Sies, 2015). Oxidative stress has been linked to diseases of the nervous system (Liu et al., 2015) and cardiovascular system (Madamanchi and Runge, 2013); aging (Stefanatos and Sanz, 2018); and neurologic hearing loss (Someya et al., 2010; Chen et al., 2015; Esterberg et al., 2016) caused by aminoglycoside-induced damage (Cheng et al., 2005) and apoptosis of hair cells from the base to the apex of the organ of Corti (Jiang et al., 2016). Aminoglycosides exert this effect by stimulating ROS production in hair cells (Mangiardi et al., 2004; Coffin et al., 2013). Mammalian hair cells are not regenerated; as such, research on therapeutic interventions for hearing loss has focused the regulation of genes responsible for hair cell proliferation and differentiation, as well as stem cell therapy (Chen et al., 2019; Czajkowski et al., 2019).

Sirtuin (SIRT)3 is a member of the Sirtuin family of NAD^+^-dependent class III histone deacetylases and/or protein ADP-ribosyl transferases that mediates adaptive responses to a variety of stressors. SIRT3 regulates mitochondrial function (Salvatori et al., 2017) and inhibits ROS production in cochlear tissue, and protects against ototoxicity (A pharmacological adverse reaction that affects the inner ear or auditory nerve, characterized by cochlear or vestibular dysfunction) induced by the aminoglycoside antibiotic gentamicin (Finkel et al., 2009; Quan et al., 2015). It was recently reported that SIRT3 can prevent hair cell apoptosis by inhibiting ROS production (Brown et al., 2014; Quan et al., 2015). Peroxisome proliferator activated receptor γ coactivator (PGC)-1α, a regulator of SIRT3 (Palacios et al., 2009; Wang et al., 2015; Li et al., 2016; Song et al., 2017), stabilizes mitochondria and regulates fatty acid oxidation and glucose metabolism (Cheng et al., 2018). PGC-1α can suppress ROS production and protect nerve cells from oxidative stress-induced damage by stimulating mitochondrial biosynthesis, enhancing the activity of the electron transport chain, and inducing the expression of a variety of ROS-detoxifying enzymes (Zhang Y. et al., 2018).

Antioxidant compounds have been investigated for their potential to protect hair cells from injury (Ylikoski et al., 2002; Tabuchi et al., 2007; Dong et al., 2015; Quan et al., 2015), but most have adverse effects, which limit their clinical applicability. As the most abundant natural flavonoid in rattan tea, dihydromyricetin (DHM) has wide-ranging pharmacologic properties, with demonstrated cardioprotective, antidiabetic, antitumor, and anti-inflammatory effects (Zhang J. et al., 2018) and no adverse effects in humans (Tong et al., 2020). DHM regulates mitochondrial function via PGC-1α in skeletal muscle and functions as an antioxidant (Zou et al., 2014); it was shown to regulate blood lipid and lipoprotein levels and protect PC12 cells from methylglyoxal-induced toxicity and endothelial cells from oxidative damage by eliminating ROS (Le et al., 2016). However, the mechanism underlying the latter effect is not well understood.

We speculated that the antioxidant property of DHM can protect against gentamicin-induced ototoxicity. To test this hypothesis, we established aminoglycoside-induced ototoxicity models using House Ear Institute-Organ of Corti (HEI-OC)1 auditory cells and cochlear explant cultures prepared from mice, and examined whether DHM pretreatment could reduce oxidative stress and apoptosis in these models. We also evaluated the role of PGC-1α and SIRT3 in the protective effects of DHM.

## Materials and Methods

### Ethics Statement

The study was approved by the Institutional Animal Care and Use Committee of China Medical University. All animals were treated in accordance with institutional guidelines.

### Cell Culture and Reagents

The HEI-OC1 auditory cell line is a widely used *in vitro* model for evaluating the ototoxicity as well as the protective effects of various agents (Jadidian et al., 2015; Kalinec et al., 2016; Park et al., 2016). HEI-OC1 cells were obtained from House Ear Institute (Los Angeles, CA, United States) and cultured in high-glucose Dulbecco’s modified Eagle’s medium (DMEM) supplemented with 10% fetal bovine serum at 33°C and 5% CO_2_. All cell culture reagents were purchased from NEST Biotech (Wuxi, China).

### Cell Viability Assay

Cell viability was assayed using 3-(4,5-dimethyl-2-thiazoyl)-2,5-diphenyltetrazoliumbromide (MTT) (Roche, Basel, Switzerland) according to the manufacturer’s instructions. HEI-OC1 cells were cultured in 96-well plates and incubated with gentamicin for 24 or 48 h before treatment with 10, 100, or 1,000 μM DHM for 24 h. MTT (0.5 mg/ml) was then added to each well for 4 h, followed by overnight incubation with lysis buffer. Optical density at 570 nm was measured using a microplate reader (Bio-Rad, Hercules, CA, United States).

### Cochlear Explant Cultures

Cochlear explant cultures were prepared from postnatal day 3 Kunming mice (Changsheng Biological Co, Liaoning Institutes for Biological Science, Shenyang, China). All animal procedures were carried out in accordance with the guidelines of the Institutional Animal Care and Use Committee of China Medical University. Before cochlea dissection, 10-mm round glass coverslips in 4-well dishes were coated with 0.012 mg/ml rat tail tendon collagen type I (Solarbio, Beijing, China). The collagen gel was allowed to solidify in air for several minutes. Cochlear explants were placed on the coverslips with DMEM-F12 (∼100 μl/dish). To evaluate whether DHM protects hair cells from gentamicin-induced ototoxicity, the cultures were divided into three groups: explants were maintained in normal DMEM-F12 for 36 h; maintained in normal medium for 24 h and then treated with 0.5 mM gentamicin for 12 h; or pretreated with DHM for 24 h followed by 0.5 mM gentamicin in the presence of DHM for 12 h.

### Immunofluorescence Analysis

Cultured cochlear explants were fixed with 4% paraformaldehyde for 15 min and washed three times for 15 min each in phosphate-buffered saline (PBS). They were then blocked for 1 h in PBS containing 0.1% Triton X-100, 5% donkey serum, and 1% bovine serum albumin (BSA). The explants were incubated overnight at 4°C with anti-myosin-VIIa antibody (1:500; Proteus Biosciences, Ramona, CA, United States), followed by Alexa Fluor 488-conjugated secondary antibody (1:200; Abcam, Cambridge, MA, United States) and Alexa Fluor 546 phalloidin (1:1000; Thermo Fisher Scientific, Waltham, MA, United States) to label hair cells; they were then examined by confocal microscopy (LSM 880; Zeiss, Oberkochen, Germany). Photographs were taken from the same area of tissue in each group (middle turn of the cochlea). The number of hair cells that were immunopositive for myosin VIIa antibody and phalloidin were counted.

### Real-Time (RT)-PCR

Total RNA was extracted from 6 cochlear explants per group using TRIzol reagent (Tiangen Biotech, Beijing, China). First-strand cDNA was synthesized using reverse transcriptase (Tiangen Biotech), and RT-PCR was performed using the SuperReal PreMix Plus kit (Tiangen Biotech) on an ABI7500 Real-time PCR system (Applied Biosystems, Foster City, CA, United States). The forward and reverse primers used in this study were synthesized by Takara Bio (Beijing, China) and had the following sequences: glyceraldehyde 3-phosphate dehydrogenase, 5′-AAATGGTGAAGGTCGGYGYGAAC-3′ and 5′-CAACAATCTCCACTTTGCCACTG-3′; PGC-1α, 5′-ACCG CAATTCTCCCTTGTATG-3′ and 5′-CTTCTGCCTCTCTCT CTGTTTGG-3′; and SIRT3, 5′-ATGCACGGTCTGTCGAA GGTC-3′ and 5′-AGAACACAATGTCGGGTTTCACAA-3′.

### Western Blotting

Cultured HEI-OC1 cells were washed three times with PBS and lysed on ice using ice-cold radioimmunoprecipitation buffer for 30 min (Millipore, Billerica, MA, United States). Protein concentration was determined using a bicinchoninic acid assay kit (Beyotime, Shanghai, China). Proteins were resolved by SDS-PAGE and transferred to a polyvinylidene difluoride membrane that was blocked with 5% BSA for 2 h and incubated overnight at 4°C with rabbit anti-SIRT3 (1:1000) and anti-PGC-1α (1:1000) antibodies (both from Affinity Biologicals, Ancaster, ON, Canada). After washing three times with Tris-buffered saline containing 0.1% Tween-20, the membrane was incubated for 2 h at room temperature with secondary antibody (1:10000; Absin Biotechnology, Shanghai, China). Anti-Bax (1:2000; Abcam, United States, anti-Bcl-2 (1:2000; Abcam, United States), cleaved PARP (1:1000; Abcam, United States) and cleaved caspase-3 (1:1000; Abcam, United States) also did the same. Protein bands were visualized with enhanced chemiluminescence reagent (Affinity Biologicals). The experiment was repeated three times. Protein levels were quantified using ImageJ software (National Institutes of Health, Bethesda, MD, United States) by analyzing gray values.

### Drug Treatment

Ototoxicity models were established in HEI-OC1 cells and cochlear explants by treatment for 12 h with 500 μM gentamicin (Meilun Biotechnology, Dalian, China). To evaluate the effects of DHM, samples were pretreated with 1 mM DHM (Meilun Biotechnology) for 24 h before adding gentamicin. To evaluate the role(s) of PGC-1α and SIRT3 in the protective effect of DHM against gentamicin-induced ototoxicity, samples were pretreated with the PGC-1α and SIRT3 inhibitors SR-18292 (20 μM; MedChemExpress, Princeton, NJ, United States) and 3-(1*H*-1,2,3-triazol-4-yl) pyridine (3-TYP, 50 μM; Absin Biotechnology), respectively, for 2 h before the addition of DHM and exposure to gentamicin.

### Flow Cytometry

Cells were stained with 2′-7′ dichlorofluorescin diacetate (DCFH-DA) solution (Abcam). Cells were incubated for 1 h at 37°C, followed by 2 washes with PBS for 5 min each. We used a FACSCalibur flow cytometer (BD Biosciences, Franklin Lakes, NJ, United States) to measure ROS levels. The fraction of apoptotic HEI-OC1 cells was quantitated by flow cytometry after staining with annexin V-fluorescein isothiocyanate (FITC)/propidium iodide (PI) (Sigma-Aldrich, St. Louis, MO, United States). The cells were seeded in 6-well culture plates and incubated with 1 mM DHM for 24 h, followed by application of 500 μM gentamicin for 12 h. Control cells were treated with culture medium containing vehicle (Cells without treatment). The cells were collected and washed with PBS and resuspended in 500 ml of 1 × binding buffer, transferred to fluorescence-activated cell sorting tubes, and stained with annexin V in accordance with the protocol for the apoptosis detection kit (Sigma-Aldrich). After incubation for 15 min at room temperature, apoptotic cells were detected by flow cytometry. Data were analyzed with Prism software (GraphPad, La Jolla, CA, United States). The experiment was repeated three times.

### Terminal Deoxynucleotidyl Transferase dUTP Nick End Labeling (TUNEL) Assay

Cultured HEI-OC1 cells were washed once with PBS and fixed with 4% paraformaldehyde for 30 min. After washing with PBS, the cells were incubated for 5 min with PBS containing 0.3% Triton X-100. After two washes with PBS, 50 μl TUNEL detection solution (Beyotime) was added to each well, and samples were incubated at 37°C in the dark for 1 h. After three washes with PBS, the samples were mounted with anti-fade solution (Beyotime) and observed under a fluorescence microscope (E800; Nikon, Japan). Cells from 10 visual fields from the same part of each of the three slides per sample were counted and averaged. Data were analyzed using Prism software.

### Quantitative Analysis

The normality of the data was confirmed with the K-S test and Q-Q figure test using SPSS software (SPSS Inc., Chicago, IL, United States). Differences between group means were evaluated by one-way analysis of variance followed by Tukey’s multiple comparisons test, and a *P* value <0.05 was considered statistically significant.

## Results

### DHM Protects Against Gentamicin-Induced Ototoxicity in HEI-OC1 Cells and Cochlear Explants

**FIGURE 1:**
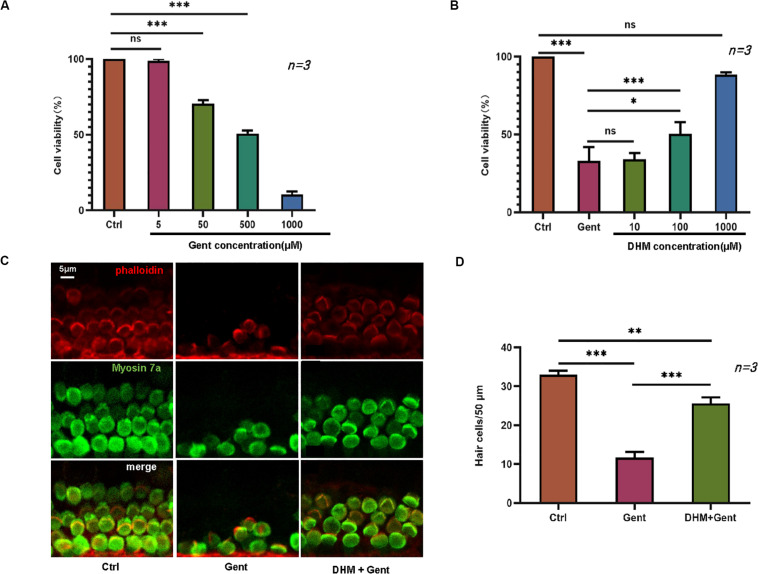
DHM protects against gentamicin-induced ototoxicity in HEI-OC1 cells and cochlear explants. **(A)** HEI-OC1 cells treated with increasing concentrations of gentamicin (0, 5, 50, and 500 μM) for 12 h. **(B)** Effect of DHM pretreatment at 0, 10, 100, and 1,000 μM for 24 h on the survival of HEI-OC1 cells exposed to 500 μM gentamicin, measured with the MTT assay. **(C,D)** Cochlear explants were divided into three groups: Ctrl (no treatment), Gent (no treatment for 24 h and 500 μM gentamicin for 12 h), and DHM + Gent (1 mM DHM pretreatment for 24 h and 500 μM gentamicin for 12 h). Samples were labeled with myosin VIIa (green) and phalloidin (red); merged images obtained by confocal microscopy are shown. Scale bar, 5 μm. Experiments were repeated three times. NS, not significant; ^∗^*P* < 0.05, ^∗∗^*P* < 0.01, ^∗∗∗^*P* < 0.001 (one-way analysis of variance followed by Tukey’s multiple comparisons test).

We first established an *in vitro* gentamicin-induced ototoxicity model by exposing HEI-OC1 cells to increasing concentrations of gentamicin (0, 5, 50, and 500 μM) for 12 h. The viability of HEI-OC1 cells was reduced in a dose-dependent manner by gentamicin treatment (Figure 1A), confirming that the ototoxicity model was successfully established. To evaluate the protective effect of DHM against gentamicin-induced toxicity, cells were treated with 500 μM gentamicin to cause substantial cell damage (∼50% reduction in viability) along with pre-treatment of 10, 100, or 1,000 μM DHM. Unexpectedly, there was no obvious improvement in viability at DHM concentrations below 100 μM; treatment with 1,000 μM DHM and 500 μM gentamicin yielded cell numbers that were comparable to the control group (Figure 1B), demonstrating that DHM abrogates the cytotoxic effects of gentamicin in a dose-dependent manner.

We also carried out a similar experiment using mouse cochlear explants cultured in regular medium or in medium supplemented with 500 μM gentamicin alone or in combination with 1,000 μM DHM, after determining the optimal concentrations of the 2 agents. Stereociliary bundles of hair cells were identified by phalloidin staining and myosin VIIa immunolabeling. In explants cultured in normal medium, there was a single row of inner hair cells and three rows of outer hair cells with polarized hair bundles protruding from the apical surface of the cell body (Figure 1C). In explants exposed to gentamicin, most hair cells were damaged and immunonegative for myosin VIIa and phalloidin staining; even the few positive cells were disorganized and fragmented. In contrast, in explants treated with both gentamicin and DHM, most hair cells were intact, with only slight disorganization of stereocilia. These observations were confirmed by quantification of surviving hair cell numbers in each group (Figure 1D), which indicated that DHM acts as an anti-ototoxic agent.

### DHM Decreases Gentamicin-Induced HEI-OC1 Cell Apoptosis

**FIGURE 2:**
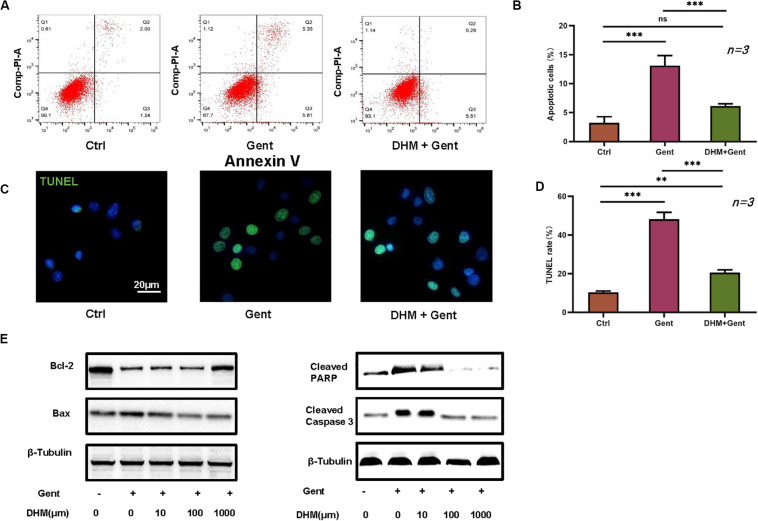
DHM attenuates gentamicin-induced HEI-OC1 cell apoptosis. **(A,B)** HEI-OC1 cells were divided into three groups: Ctrl (no treatment), Gent (500 μM gentamicin treatment for 12 h), and DHM + Gent (1,000 μM DHM pretreatment for 24 h and gentamicin treatment for 12 h). Apoptotic cells were detected by flow cytometry. **(C,D)** Quantitative analysis of TUNEL-positive cells. Scale bar, 20 μm. **(E)** Western blot analysis of B-cell lymphoma 2-associated X protein (Bax), cleaved caspase-3, and cleaved PARP expression as a function of DHM concentration. β-Tubulin was used as a loading control. Experiments were repeated three times. NS, not significant; ^∗∗^*P* < 0.01, ^∗∗∗^*P* < 0.001 (1-way analysis of variance followed by Tukey’s multiple comparisons test).

We evaluated the effect of DHM on HEI-OC1 cell apoptosis by flow cytometry and with the TUNEL assay. Dead cells were labeled by PI and those undergoing apoptosis were labeled by annexin V. The proportion of apoptotic cells was increased after gentamicin treatment compared to the control group; however, the effect was reversed by pretreatment with DHM compared to the gentamicin-only group (Figures 2A,B). Additionally, while few TUNEL-positive cells were detected in the control group, numerous cells were observed following gentamicin treatment; the number was reduced by DHM pretreatment (Figures 2C,D). Western blot analysis revealed the downregulation of B-cell lymphoma 2-associated X protein, cleaved PARP, and cleaved caspase-3 over time following DHM pretreatment relative to control cells (Figure 2E).

### DHM Alleviates Gentamicin-Induced Oxidative Stress in HEI-OC1 Cells

**FIGURE 3:**
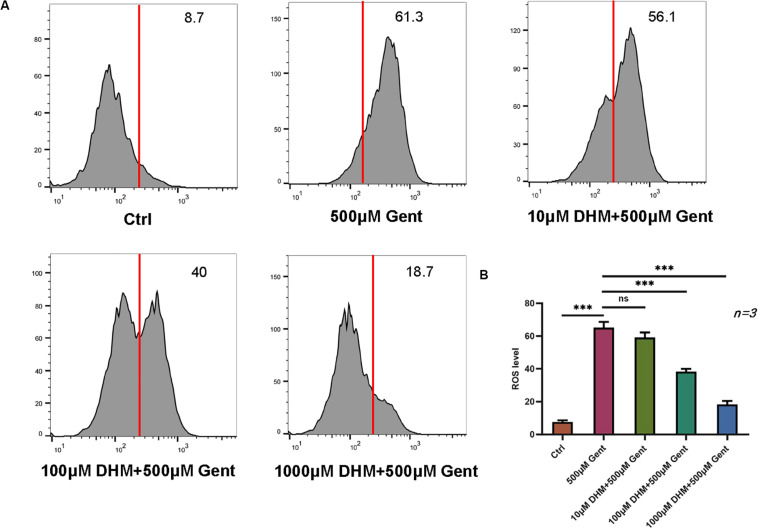
DHM attenuates gentamicin-induced oxidative stress in HEI-OC1 cells. **(A,B)** HEI-OC1 cells were incubated with increasing concentrations of DHM. ROS levels after gentamicin treatment with or without DHM pretreatment were evaluated by DCFH-DA staining combined with flow cytometry. The experiment was repeated three times. NS, not significant; ^∗∗∗^*P* < 0.001 (one-way analysis of variance followed by Tukey’s multiple comparisons test).

Given that gentamicin-induced hair cell apoptosis is mediated by ROS (Yang et al., 2011), we investigated the effect of DHM on ROS levels in HEI-OC1 cells by flow cytometry. The ototoxicity model was established with a gentamicin concentration of 500 μM. The results showed that ROS levels were increased after gentamicin treatment compared to control cells; the levels were reduced in the presence of increasing concentrations of DHM starting at 100 μM (Figures 3A,B).

### Protection Against Gentamicin-Induced Hair Cell Damage by DHM Involves PGC-1α and SIRT3

**FIGURE 4:**
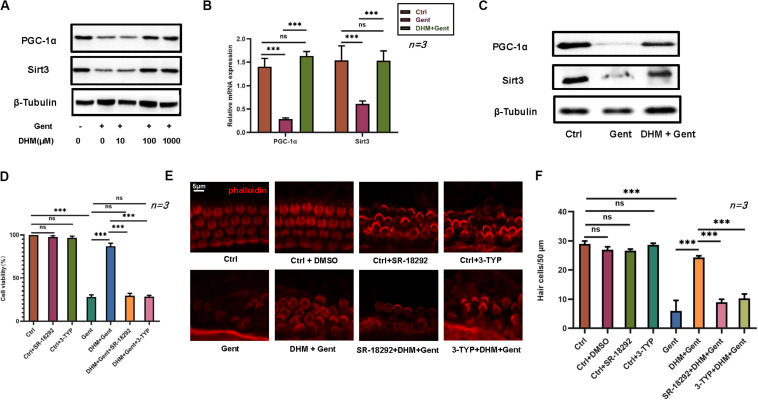
PGC-1α and SIRT3 mediate the protective effect of DHM against gentamicin-induced hair cell damage. **(A)** Endogenous PGC-1α and SIRT3 protein expression in HEI-OC1 cells as determined by western blotting; β-tubulin was used as a loading control. **(B)** RT-PCR detection of PGC-1α and SIRT3 transcript levels in cochlear explants after gentamicin treatment with or without DHM (1 mM) pretreatment; glyceraldehyde 3-phosphate dehydrogenase (GAPDH) was used as an internal control. **(C)** Western blot analysis of PGC-1α and SIRT3 protein expression after DHM (1 mM) pretreatment; β-tubulin was used as a loading control. **(D–F)** Effect of PGC-1α and SIRT3 inhibition (with SR-18292 and 3-TYP, respectively) on the viability of HEI-OC1 cells **(D)** and hair cells in cochlear explants **(E,F)** treated with gentamicin with or without DHM pretreatment. Experiments were repeated three times NS, not significant; ^∗∗∗^*P* < 0.001 (one-way analysis of variance followed by Tukey’s multiple comparisons test).

We investigated the mechanisms underlying the effects of DHM by evaluating PGC-1α and SIRT3 levels by RT-PCR and western blotting. Expression of PGC-1α and SIRT3 proteins in HEI-OC1 cells markedly increased with DHM concentration (Figure 4A). In cochlear explants, pretreatment with DHM for 24 h prior to gentamicin application increased both mRNA and protein levels of PGC-1α and SIRT3 compared to samples treated with gentamicin only (Figures 4B,C). To confirm the involvement of PGC-1α and SIRT3 in the protective effects of DHM, we treated HEI-OC1 cells and cochlear explants with pharmacologic inhibitors of PGC-1α (SR-18292) and SIRT3 (3-TYP) prior to DHM pretreatment and exposure to gentamicin (Figures 4D–F). Inhibiting PGC-1α and SIRT3 attenuated the effects of DHM without altering the survival of HEI-OC1 cells, although some deformation of hair cell cilia was observed.

**FIGURE 5:**
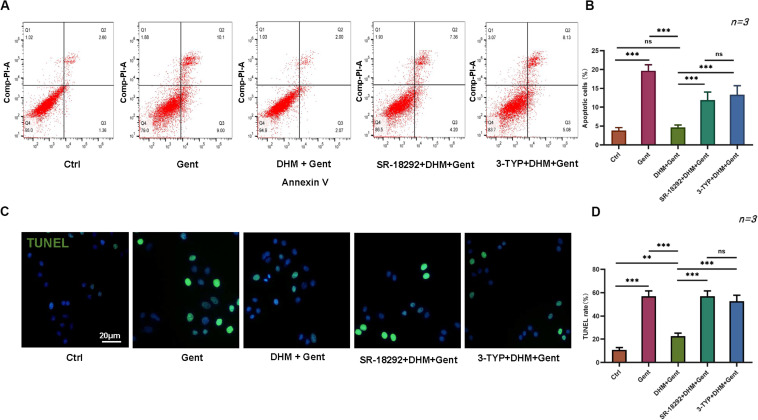
PGC-1α and SIRT3 mediate the protective effect of DHM against gentamicin-induced HEI-OC1 cell apoptosis. **(A,B)** Flow cytometry detection of apoptosis in HEI-OC1 cells treated with PGC-1α and SIRT3 inhibitors (SR-18292 and 3-TYP, respectively) prior to DHM and gentamicin treatment. **(C,D)** Quantitative analysis of TUNEL-positive cells following pretreatment with 20 μM SR-18292 and 50 μM 3-TYP for 2 h prior to 1 mM DHM treatment and gentamicin exposure. Experiments were repeated three times. NS, not significant; ^∗∗^*P* < 0.01, ^∗∗∗^*P* < 0.001 (one-way analysis of variance followed by Tukey’s multiple comparisons test).

To examine the functions of PGC-1α and SIRT3 in greater detail, HEI-OC1 cells were treated with SR-18292 and 3-TYP and the fraction of apoptotic cells was evaluated by flow cytometry and with the TUNEL assay. Inhibitor pretreatment increased the number of apoptotic cells compared to the DHM + gentamicin group (Figures 5A,B); the same trend was observed by quantifying the number of TUNEL-positive cells (Figures 5C,D). These results indicate that PGC-1α and SIRT3 are involved in the protective effects of DHM in hair cells.

### The Antioxidant Effect of DHM in HEI-OC1 Cells Is Mediated by PGC-1α and SIRT3

**FIGURE 6:**
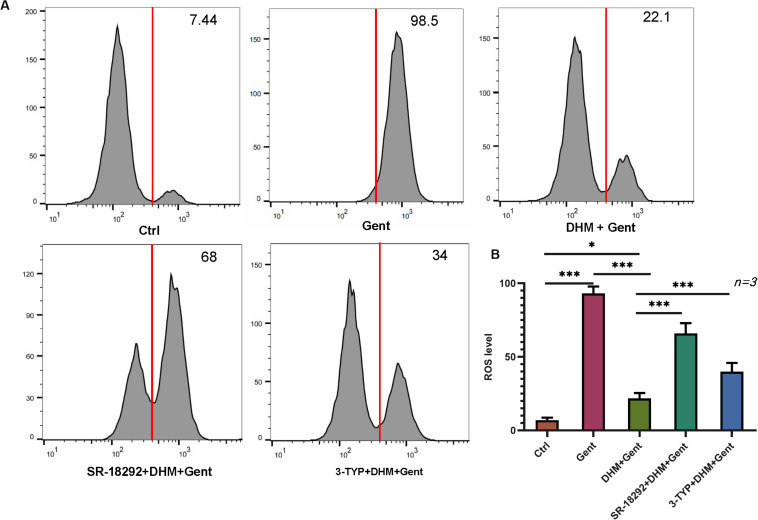
PGC-1α and SIRT3 mediate the protective effect of DHM against gentamicin-induced oxidative stress in HEI-OC1 cells. **(A,B)** HEI-OC1 cells were pretreated with PGC-1α and SIRT3 inhibitors (SR-18292 and 3-TYP, respectively) before treatment with 1 mM DHM followed by 500 μM gentamicin for 12 h. Intracellular ROS levels were measured by DCFH-DA staining combined with flow cytometry. The experiment was repeated three times. ^∗^*P* < 0.05, ^∗∗∗^*P* < 0.001 (one-way analysis of variance followed by Tukey’s multiple comparisons test).

The above results showed that DHM inhibits apoptosis in HEI-OC1 cells induced by gentamicin by suppressing ROS production. To determine whether this involves PGC-1α and SIRT3, SR-18292 and 3-TYP were added to HEI-OC1 cell cultures, prior to DHM and gentamicin treatment; ROS levels were then assessed by DCFH-DA staining and flow cytometry. We found that PGC-1α and SIRT3 inhibition markedly increased ROS levels compared to cells without SR-18292 and 3-TYP treatment (Figure 6). Thus, DHM prevents ototoxicity via the PGC-1α/SIRT3 axis.

### PGC-1α Regulates SIRT3 Expression in HEI-OC1 Cells and Cochlear Explants

**FIGURE 7:**
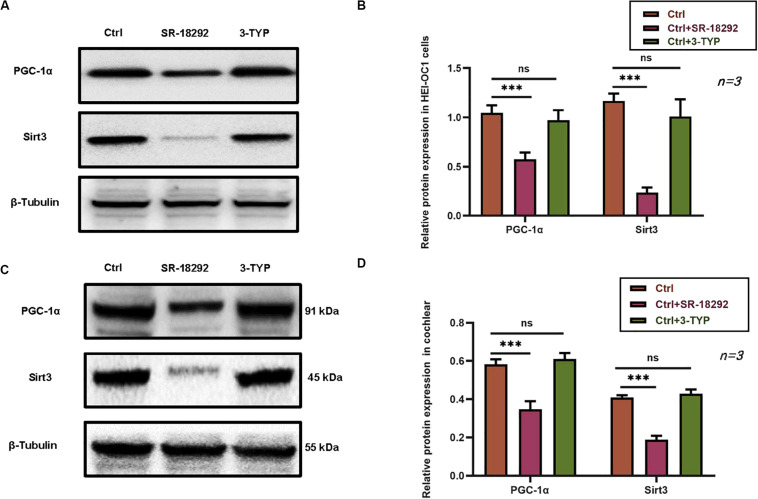
PGC-1α regulates SIRT3 expression in HEI-OC1 cells and cochlear explants. **(A–D)** Effect of pretreatment with the PGC-1α inhibitor SR-18292 and SIRT3 inhibitor 3-TYP on PGC-1α and SIRT3 protein levels in HEI-OC1 cells **(A,B)** and cochlear explants **(C,D)**, as determined by western blotting. The experiment was repeated three times. NS, not significant; ^∗∗∗^*P* < 0.001 (one-way analysis of variance followed by Tukey’s multiple comparisons test).

The relationship between PGC-1 α and SIRT3 in the auditory system has not been previously reported. We therefore investigated the expression of PGC-1α signaling pathway components, including SIRT3. Treatment with the PGC-1α inhibitor SR-18292 resulted in the downregulation of PGC-1α and SIRT3, whereas SIRT3 inhibition by 3-TYP application had no effect on the protein expression of PGC-1α (Figures 7A,B). Similar results were obtained in cochlear explants (Figures 7C,D). Thus, DHM exerts its protective against gentamicin-induced ototoxicity via PGC-1α/SIRT3 signaling, with PGC-1α acting as an upstream regulator of SIRT3 expression.

## Discussion

Aminoglycosides can induce excessive ROS production in cells; this activates caspase-dependent and -independent apoptosis signaling cascades (Chan, 2005; Genestra, 2007) and leads to ototoxicity (Esterberg et al., 2016; Sheth et al., 2017; Desa et al., 2018; Guo et al., 2018) and hearing loss. From a clinical standpoint, it is important to identify new therapeutic strategies to prevent or treat ototoxicity caused by aminoglycoside antibiotics.

DHM exerts antioxidant effects via multiple signaling pathways including AMP-activated protein kinase (AMPK), mitogen-activated protein kinase, nuclear factor E2-related factor 2, ATP-binding cassette transporter A1, and peroxisome proliferator-activated receptor γ (Zhang J. et al., 2018). DHM was shown to suppress ROS production and H_2_O_2_-induced apoptosis in human pulmonary arterial smooth muscle cells (Hou et al., 2015). In the present study, we established ototoxicity models using HEI-OC1 cells and mouse cochlear explants and investigated whether DHM can protect against hair cell injury caused by gentamicin. We found that DHM pretreatment improved HEI-OC1 cell viability and the survival of hair cells in cochlear explants, and determined that the underlying mechanism involves inhibition of apoptosis via suppression of ROS production mediated by PGC-1α/SIRT3 signaling.

The cytoprotective effects of DHM in a variety of pathologic conditions including non-alcoholic fatty liver disease (NAFLD) (Zeng et al., 2019), osteoarthritis (Wang et al., 2018), cardiac ischemia/reperfusion injury (Wei et al., 2019), and hypobaric hypoxia-induced memory impairment involve the modulation of SIRT3 expression and/or activity. SIRT3 has been reported to protect hair cells against gentamicin-induced ototoxicity by stimulating the conversion of NADP^+^ to NADPH in mitochondria, thereby suppressing ROS production (Quan et al., 2015). Accordingly, we observed that SIRT3 protein expression was enhanced by pretreatment with DHM, which has known antioxidant effects. However, the mechanism by which DHM regulates SIRT3 has not been reported. In previous studies, DHM was found to increase the expression of PGC-1α in skeletal muscle (Zhou et al., 2015; Huang et al., 2018). PGC-1α is a transcription factor that regulates lipid metabolism by inducing the expression of multiple genes in the tricarboxylic acid cycle and mitochondrial fatty acid oxidation pathway (Cheng et al., 2018). Additionally, PGC-1α regulates mitochondrial gene expression and oxidative metabolism (Islam et al., 2018). However, the function of PGC-1α in aminoglycoside-induced ototoxcity models has never been studied. Most previous research on the regulatory relationship between PGC-1α and Sirtuin family proteins have focused on SIRT1. It was reported that DHM inhibited lipid accumulation in hepatocytes by activating AMPK/PGC-1α/estrogen related receptor α signaling to increase SIRT3 expression and reduce oxidative stress, thereby preventing the development of NAFLD (Zeng et al., 2019). Our data showed that PGC-1α and SIRT3 expression increased with DHM concentration, suggesting that the anti-ototoxic effect of DHM involves these factors. To assess this possibility, we used pharmacologic inhibitors of PGC-1 α and SIRT3 prior to DHM pretreatment. The alleviation of apoptosis and oxidative stress by DHM was abrogated by both agents, providing support for our hypothesis.

The regulatory relationship between PGC-1α and SIRT3 has been previously described (Zhang et al., 2016; Song et al., 2017; Yu et al., 2017), but not in the context of the auditory system. We determined that PGC-1α functions as a transcriptional regulator of SIRT3 expression in HEI-OC1 cells and cochlear explants, providing additional evidence that PGC-1α/SIRT3 signaling mediates the effects of DHM. However, rescue experiments are needed in future studies to validate these findings.

It is worth noting that the working concentration of DHM varies across experimental systems and tissues. In palmitic acid-treated human umbilical vein endothelial cells (HUVECs), application of 1 μM DHM for 12 h suppressed apoptosis and ROS production but in H_2_O_2_-treated HUVECs, a concentration of 300 μM was required to achieve the same effect (Tong et al., 2020). In our study, 100 μM DHM had statistically significant but mild effects on cell viability, apoptosis, and gene expression in both HEI-OC1 cells and cochlear explants; we therefore used a working concentration of 1 mM DHM and observed more robust effects. One major limitation of our study was the lack of *in vivo* experiments; therefore, it is unclear whether DHM has the same protective effects in living animals.

In conclusion, this is the first demonstration that DHM protects against gentamicin-induced ototoxicity PGC-1α/SIRT3 signaling. Our findings indicate that DHM may be effective in the prevention or treatment of hearing loss caused by aminoglycoside antibiotics as well as other oxidative stress-related diseases.

## Data Availability Statement

All datasets presented in this study are included in the article/Supplementary Material.

## Ethics Statement

This study was approved by the Institutional Animal Care and Use Committee, China Medical University, and all animals were treated in accordance with the institutional guidelines.

## Author Contributions

XM designed the study. HH performed the experiments. HH and YD analyzed the data and wrote the manuscript. All authors contributed to the article and approved the submitted version.

## Conflict of Interest

The authors declare that the research was conducted in the absence of any commercial or financial relationships that could be construed as a potential conflict of interest.
