# Supplementary material for: Dihydromyricetin Protects Against Gentamicin-Induced Ototoxicity via PGC-1α/SIRT3 Signaling in vitro
Source: Front Cell Dev Biol. 2020 Jul 28;8:702. doi: 10.3389/fcell.2020.00702 (PMC7399350; doi:10.3389/fcell.2020.00702)
Supplement: Supplementary file 1 [file Data_Sheet_1.pdf]

Supplementary Material

Supplementary Figures

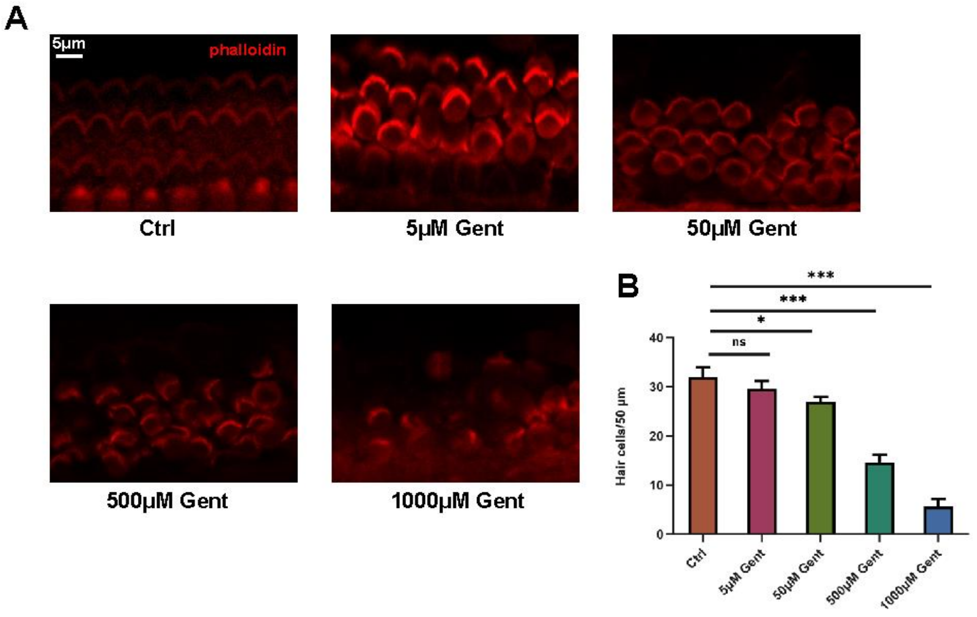

Supplementary Figure 1. Gentamicin reduces hair cell viability in cochlear explants. (A) Mouse cochlear explants were isolated and cultured. The indicated concentration of gentamicin (5, 50, 500, and 1000 μM) was applied for 12 h to the middle turn of the cochlea in each sample. Hair cells were labeled with phalloidin (red), and representative images were obtained by confocal microscopy are shown. Scale bar, 5 μm. **(B)** Quantitative analysis of results in (A). The experiment was repeated 3 times. NS, not significant; *P<0.05, ***P<0.001 (1-way analysis of variance followed by Tukey's multiple comparisons test).

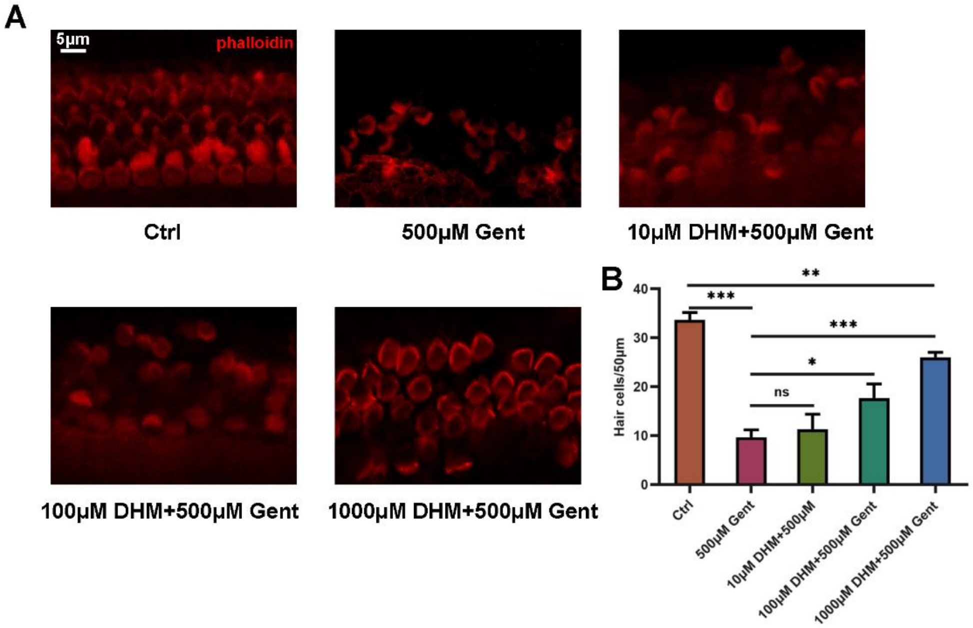

Supplementary Figure 2. DHM inhibits gentamicin-induced ototoxicity in cochlear explants. **(A)** Cochlear explants were divided into 5 treatment groups (control; 500 µM gentamicin; 10 µM DHM+500 µM gentamicin; 100 µM DHM+500 µM gentamicin; 1000 µM DHM+500 µM gentamicin). DHM was added to the culture 24 h before gentamicin. Hair cells were labeled with phalloidin (red), and representative images obtained by confocal microscopy are shown. Scale bar, 5 µm. **(B)** Quantitative analysis of results in (A). The experiment was repeated 3 times. NS, not significant; *P<0.05, ***P<0.001 (1-way analysis of variance followed by Tukey’s multiple comparisons test).

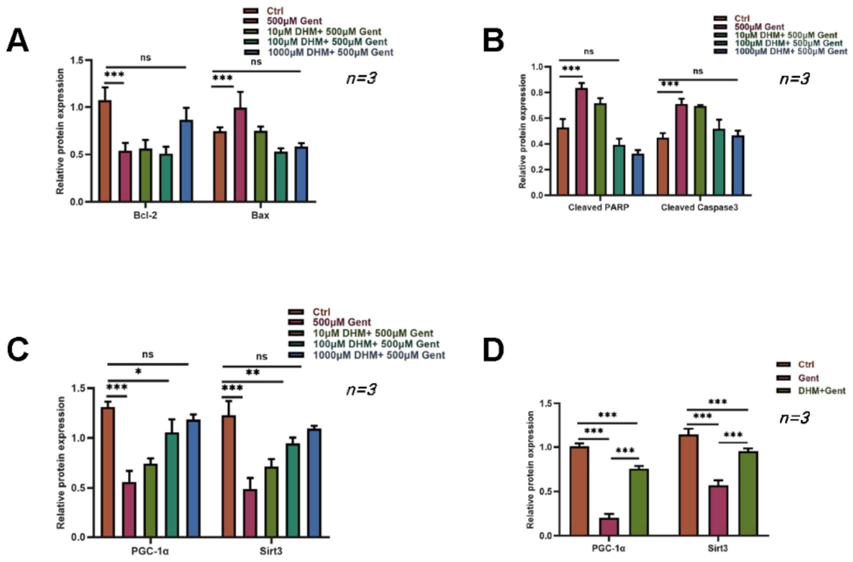

Supplementary Figure 3. Quantification of apoptosis-related protein expression in HEI-OC1 cells and cochlear explants. (A, B) B-cell lymphoma (Bcl)-2, Bcl-2-associated X protein (Bax), cleaved PARP, and cleaved caspase-3 protein levels detected by western blotting. **(C)** PGC-1α and SIRT3 protein expression in HEI-OC1 cells pretreated with increasing concentrations of DHM. **(D)** PGC-1α and SIRT3 protein expression in cochlear explants with/without DHM pretreatment. Experiments were repeated 3 times. NS, not significant; *P<0.05, ***P<0.001 (1-way analysis of variance followed by Tukey's multiple comparisons test).

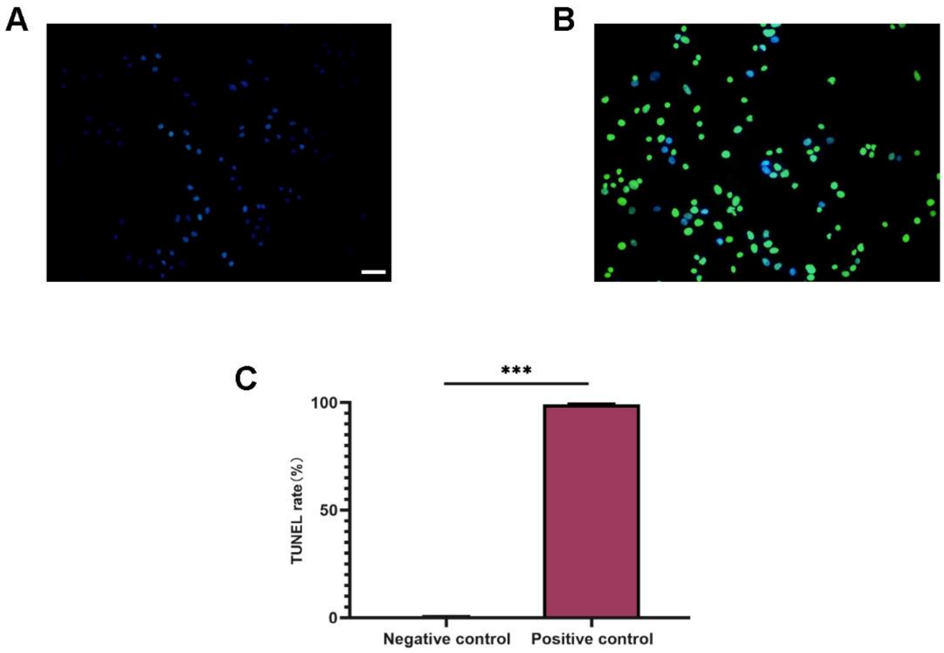

Supplementary Figure 4. Negative and positive controls for the TUNEL assay. (A) TUNEL staining was undertaken on HEI-OC1 cells cultured in normal medium without gentamicin and DHM treated. Subsequent steps are carried out according to standard TUNEL staining. **(B)** TUNEL staining was undertaken on HEI-OC1 cells cultured with DNase I (1:100 dilution). Subsequent steps are carried out according to standard TUNEL staining. **(C)** Quantification of TUNEL-positive cells in (A, B). Scale bar, 50 μm. Experiments were repeated 3 times. NS, not significant; *$P<0.05$, ***$P<0.001$ (1-way analysis of variance followed by Tukey’s multiple comparisons test).
